# Application of Synchronous Text-Based Dialogue Systems in Mental Health Interventions: Systematic Review

**DOI:** 10.2196/jmir.7023

**Published:** 2017-07-21

**Authors:** Simon Hoermann, Kathryn L McCabe, David N Milne, Rafael A Calvo

**Affiliations:** ^1^ Positive Computing Laboratory School of Electrical and Information Engineering The University of Sydney Sydney Australia; ^2^ Psychiatry and Behavioral Sciences University of California Davis, CA United States

**Keywords:** chat, dialog system, remote psychotherapy

## Abstract

**Background:**

Synchronous written conversations (or “chats”) are becoming increasingly popular as Web-based mental health interventions. Therefore, it is of utmost importance to evaluate and summarize the quality of these interventions.

**Objective:**

The aim of this study was to review the current evidence for the feasibility and effectiveness of online one-on-one mental health interventions that use text-based synchronous chat.

**Methods:**

A systematic search was conducted of the databases relevant to this area of research (Medical Literature Analysis and Retrieval System Online [MEDLINE], PsycINFO, Central, Scopus, EMBASE, Web of Science, IEEE, and ACM). There were no specific selection criteria relating to the participant group. Studies were included if they reported interventions with individual text-based synchronous conversations (ie, chat or text messaging) and a psychological outcome measure.

**Results:**

A total of 24 articles were included in this review. Interventions included a wide range of mental health targets (eg, anxiety, distress, depression, eating disorders, and addiction) and intervention design. Overall, compared with the waitlist (WL) condition, studies showed significant and sustained improvements in mental health outcomes following synchronous text-based intervention, and post treatment improvement equivalent but not superior to treatment as usual (TAU) (eg, face-to-face and telephone counseling).

**Conclusions:**

Feasibility studies indicate substantial innovation in this area of mental health intervention with studies utilizing trained volunteers and chatbot technologies to deliver interventions. While studies of efficacy show positive post-intervention gains, further research is needed to determine whether time requirements for this mode of intervention are feasible in clinical practice.

## Introduction

Web-based technologies offer novel ways to deliver mental health interventions that, according to the literature, can be effective [1-3]. One form of online psychological intervention is synchronous text-based, one-to-one conversation (or “chat”) in its different incarnations: SMS text messaging (short message service, mobile phone apps, or websites.

Previous systematic reviews by Barak and colleagues [1], as well as by Dowling and Rickwood [4], evaluated psychological interventions delivered via text-based communication. Focused specifically on synchronous text-based communication, Dowling and Rickwood identified 6 studies meeting their study inclusion criteria, and they noted some evidence to support this mode of mental health intervention. However, they also reported that the small number and overall low quality of the studies indicated weak empirical support for synchronous text-based interventions. Since the writing of these reviews, new technologies have changed the landscape of such mental health interventions. In particular, the penetration of mobile phone technologies and improvements in artificial intelligence and natural language processing (NLP) techniques [5] means that with more tools available to deliver this mode of intervention, one could reasonably expect that the body of research investigating synchronous text-based online interventions have similarly evolved. In particular, the boom of messaging services among major social media companies and the more recent rally to build automated services are driving innovation in this space. Several start-ups companies have been established in this sector and have released products designed to provide mental health services using these new technologies, including, for example, 7cups.com and talklife.co. Although some of these new organizations may be carrying evaluations of their services, the constraints of the commercial world mean not much is known yet. However, the accelerated pace of innovation and uptake in this space requires a systematic evaluation and analysis of the research evidence on how these services are being applied and how well the application of these tools can support mental health and well-being. For example, determining the impact of automation (eg, application of NLP to generate text-based dialogue) on the efficacy of mental health interventions may have significant implications on the cost of providing and accessing mental health services. Studies that use sensor data or automatically generated summaries to drive conversation may also have significant impact on how mental health interventions can be delivered.

The modes used to deliver mental health support have been diversifying and are becoming increasingly technologically complex. At a simple level, chat-based texting has different incarnations: as an SMS service, a dedicated mobile app, or embedded within websites. These different methods of delivery allow for individuals to engage in one-to-one contact with a therapist, a peer, or in some cases even automated “relational agents,” where individuals converse with an artificial intelligence system [6,7]. The conversations may even use information acquired from sensors that track activity or sleep. Some of these innovations have been explored in recent systematic reviews of largely asynchronous applications of texting interventions in mental health [8] and public health more broadly. Asynchronous interventions are when clients do not have the expectation of receiving an immediate reply. These have been used to encourage medication adherence or treatment compliance, aftercare support (eg, [9-11]), deliver appointment reminders [12], and to monitor and capture mood or symptom change [13], especially in conditions with high relapse rates (eg, alcoholism and schizophrenia].

The potential benefits and limitations of synchronous text-based interventions (ie, a dialogue where an immediate reply is expected) still need to be considered. This article is part of a special issue on computing and mental health aimed at bringing both communities together. The computing literature tends to focus on feasibility studies, whereas mental health literature tends to apply better-known technologies to study intervention efficacy. Thus, there is a continuum of research studies examining synchronous text-based interventions from early feasibility studies to those that examine evidence of efficacy. The challenge of bringing these 2 bodies of literature together is addressed in the present systematic review of individual synchronous text-based psychological interventions. Thus, whereas the primary objective of this systematic review is to report studies evaluating the efficacy of such interventions, we also considered it valuable to the reader to include a summary of feasibility studies. Therefore, we provide a concise overview of the state of scientific research on synchronous text-based psychological interventions that we hope will guide researchers as well as mental health service providers to the most promising avenues for research and implementation.

## Methods

### Objective

The objective of this study was to provide a systematic review and synthesis of the application of synchronous one-to-one text-based chat and texting in psychological interventions. The focus of this systematic review is the mode of delivery of the intervention independently of the client group and their impact on mental health outcomes. For the purpose of this review, “Web-based chat” is defined as text-based communication over the Web that allows participants to rapidly exchange messages, and thereby, mimics spoken dialog (eg, text communication part of Facebook messenger, WhatsApp, or Skype). Similarly “texting” also involves the exchange of text-based messages but by using mobile devices and cellular networks for transmission (eg, SMS text messaging or MMS multimedia messaging).

**Figure 1:**
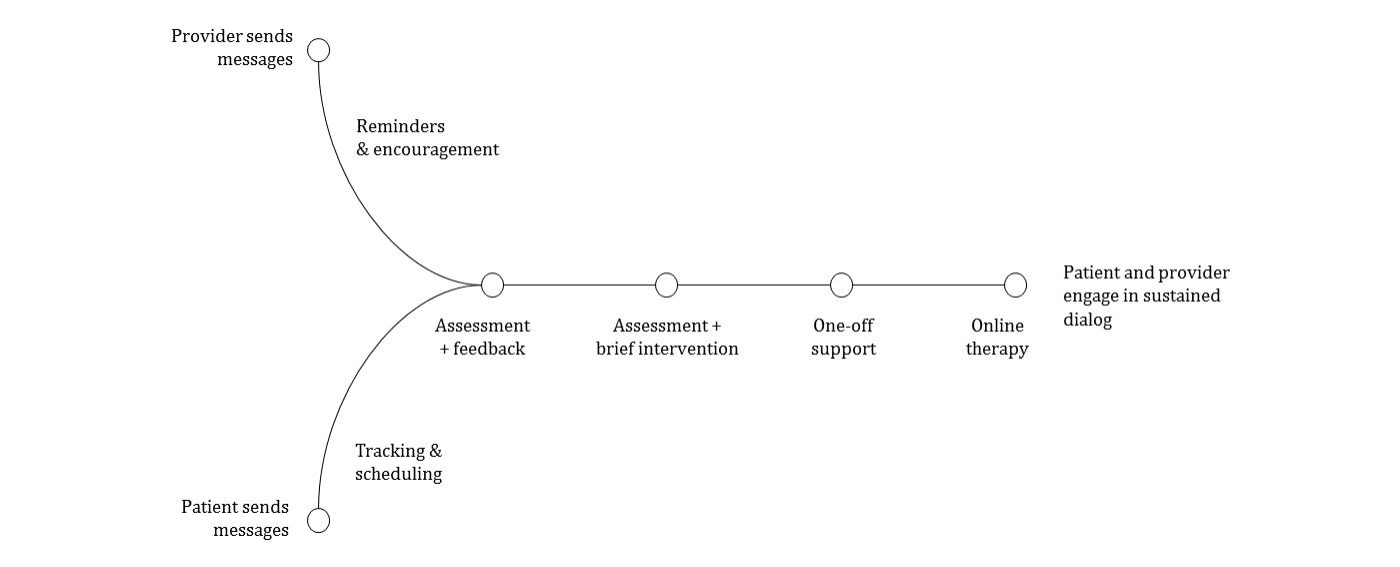
Taxonomy of the use of text-based communication in mental health interventions – spectrum from low volume (left) to high volume text-based interaction (right).

In Figure 1, we present a taxonomy to illustrate text-based communication pathways in psychological interventions. It ranges from leftmost (a) low volume communication reduced to a single message such as an appointment reminder or confirmation that augment the normal therapy to rightmost (b) high volume communication, where the entire intervention is carried out via text-based communication. For this systematic review, only studies that included at least several bidirectional message exchanges were included.

The study followed the Preferred Reporting Items for Systematic Review and Meta-Analysis Protocols guidelines [14,15]. The protocol including the exact search strategy for this systematic review was registered prospectively at PROSPERO (Registration Number: CRD420160494490).

### Data Sources and Search Strategy

The search strategy included a combination of mental health and computing terminology, as well as keywords related to the type of intervention (eg, dialogue, chat, text, and messaging), intervention, and outcomes (eg, counseling, adherence, feasibility, mood, quality of life, and mental health). Preliminary searches of Medical Literature Analysis and Retrieval System Online (MEDLINE), produced a poor match for the study, and only a limited number of studies were identified. Therefore, the search strategy was revised and terms were expanded to include a broader range of mental health conditions in order to boost the number of studies retrieved. Keywords were collected from various sources including scientific literature, mental health websites, and the Diagnostic and Statistical Manual of Mental Disorders, Fifth Edition (DSM 5). Also included were search terms referencing situations that are associated with mental health and well-being, such as bullying or cyber bullying, abuse, violence, and stigma.

The revised search strategy was then adapted for application using OvidSP on the following databases: MEDLINE (1996-October 2016), PsycINFO (1987-October 2016), EMBASE (1974-October 2016), as well as the Cochrane Central Register of Controlled Trials (September 2016). Web of Science (1996-present), Scopus (1996-present), ACM Digital Library (1996-present), and IEEE Xplore (1996-present) searches were also performed. The final search strategy was based on the three main topic areas of the review: (1) psychological interventions, (2) use of text-based dialogue, and (3) application to mental health and well-being. Searches in MEDLINE, PsyInfo, and EMBASE were carried out using the advanced search functionality and included keyword truncations and mappings to subject heading (Medical Subject Heading [MeSH] and emtree) that were adapted for each database. Similar to other systematic reviews reported in this area of research [16,17], the reference lists of all studies meeting the inclusion criteria resulting from the database were hand searched to identify all references related to the topic.

The primary focus was psychological outcomes of synchronous text-based intervention, though some technical description, caregiver, or individual evaluations of the intervention were occasionally reported. Based on the taxonomy displayed in Figure 1, we included studies reporting feasibility, adherence, or acceptability of synchronous individual text-based interventions for mental health and well-being.

### Selection Criteria

Studies were included if they were published in English and in peer-reviewed scientific journals or conference papers (common high quality publishing venues in engineering and computer science). Specific criteria relating to intervention type and outcome(s) are described below.

Included were studies where synchronous text-based dialogue was used in a psychological intervention, where text-based dialogue was the main intervention or as a control intervention in only one aspect or one arm of the study design. Broad definitions and search terms for mental health and well-being were used for inclusion criteria, all based on the Diagnostic and Statistical Manual of Mental Disorders (DSM) listed psychiatric diagnoses such as addiction (eg, alcohol and cigarette smoking). Intervention terms were also searched, including promoting resilience, motivation, and positive psychological outcomes, as well as mainstream definitions of experiences that affect mental health and well-being.

We excluded studies if the intervention was not primarily based on synchronous text-based communication, such as when the main mode of communication was spoken communication (eg, telephone) or face-to-face communication (eg, video chat and in-person therapy session). Studies were also excluded if the format was not one-on-one, such as group dialogue (eg, a psychologist moderates an online group discussion in a chat-room). Additionally, studies were excluded when the intervention outcomes did not include mental health and well-being, including when studies were limited to health behaviors (eg, weight loss, hypertension reduction, and exercise support). Studies were also excluded if the intervention reported fewer than 5 participants, or utilized unidirectional (ie, only one entity sends messages) or nonsynchronous (eg, email) communication.

Given the focus of the study was the mode of intervention (synchronous texting), the inclusion of a control condition in the study design was not a key requirement and was therefore not considered grounds for exclusion.

Studies from the database search were exported to systematic review software (Covidence) for ease of management. Duplicate records were deleted. An independent review of study titles and abstract was conducted for all studies by 2 authors (SH and KLM). Articles not meeting the stated inclusion or exclusion criteria were removed. Two authors (SH and KLM) independently conducted full text screening of studies to select the studies to be included. Finally, a reference list search of each of the studies selected from the previous step was conducted to identify additional studies. Additional studies were also independently evaluated by 2 authors (SH and KLM) against the inclusion and exclusion criteria. The same 2 authors extracted data from each study into a form based on systematic review literature. Where consensus was not established, additional reviewers (RAC and DNM) were enlisted to resolve selection disputes.

## Results

### Study Selection

**Figure 2:**
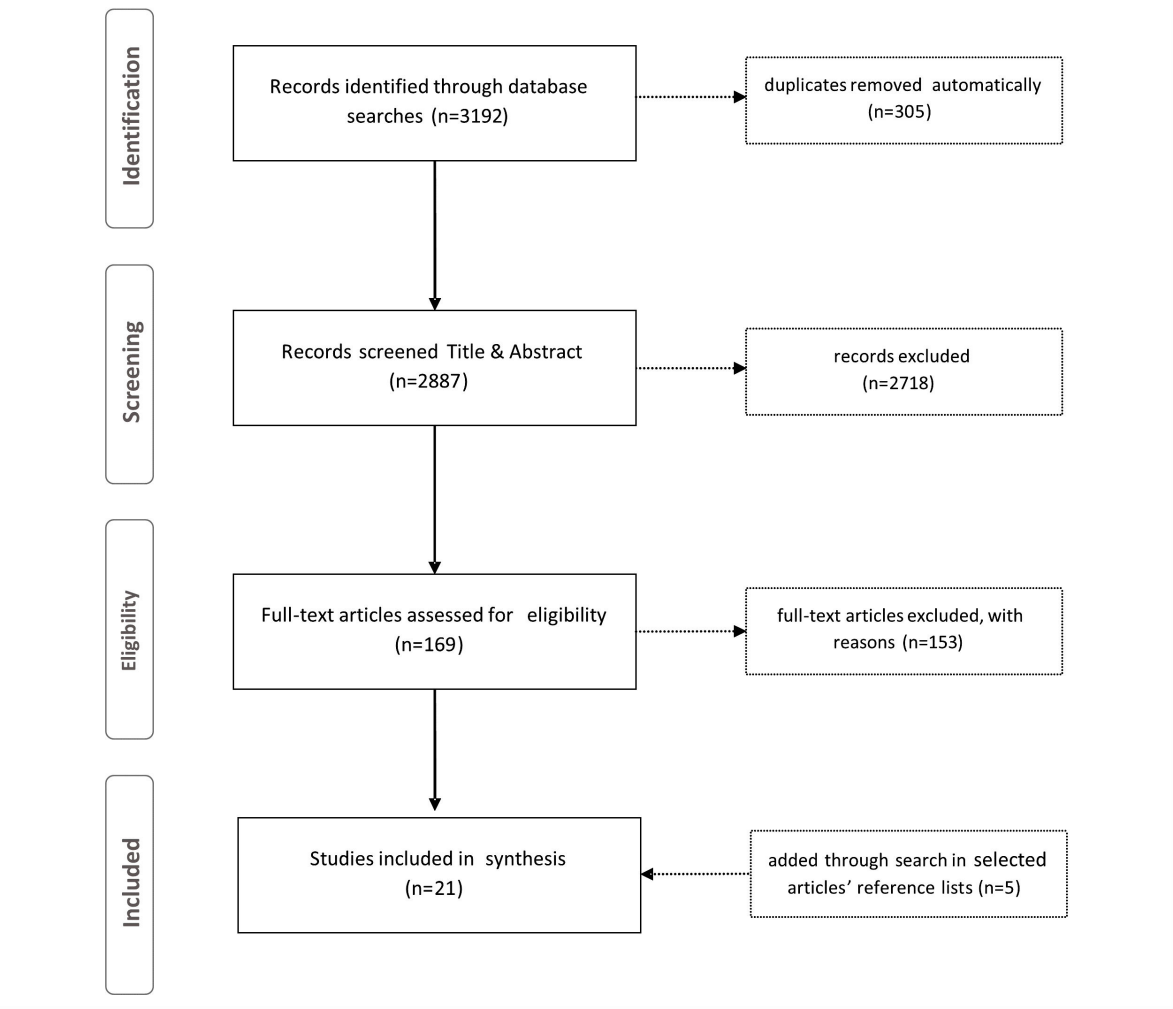
Flow of information through different phases of systematic review.

A total of 3192 articles were retrieved from the following databases: MEDLINE (n=551), PsycINFO (n=1273), EMBASE (n=329), Web of Science (n=311), Scopus (n=271), Central (n=344), IEEE Xplore (n=43), and ACM DL (n=70). After automated identification of duplicates with Endnote X7, 2887 articles remained. Title and abstract-level screening resulted in the elimination of studies that did not report a psychological intervention, did not measure efficacy as a primary outcome, did not use individualized synchronous chat, or consisted of reviews, meta-analyses, dissertation, or opinion. In-depth review of the remaining 169 articles resulted in the elimination of 153 studies. Reference lists of the remaining 16 studies were searched, and a further 5 studies were included in the review. Figure 2 shows an overview of the process. Finally, intervention technology meeting inclusion criteria was identified outside of the database search. These 3 studies have been included in the feasibility studies summary and table (Multimedia Appendices 1 and 2).

Among the retrieved studies, there was significant heterogeneity in terms of mental health conditions, psychological outcomes, intervention design, and subjective ratings of mental health (eg, mood and symptom ratings). For these reasons, the results are presented in separate tables as well as a narrative analysis. Studies reporting efficacy are described in Multimedia Appendix 3, and studies reporting acceptability or feasibility are presented separately in Multimedia Appendix 1. Additionally, a narrative analysis of studies presented in Multimedia Appendix 1 can be found in Multimedia Appendix 2.

### Efficacy Evaluation Studies

Studies S1-S14 [18-31] are described in Multimedia Appendix 3. Information (where available) is provided relating to the study: location, sample, experimental design, type of intervention, intervention duration, control or comparison condition, attrition, and the main results of the study.

In summary, the studies meeting inclusion criteria varied in the target populations, intervention sample size (eg, intervention group range: n=5-152), treatment type, treatment length, as well as specific outcome measures. The majority of studies were completed in Europe and the United Kingdom (S1, S3, S5-7, S9, S10, S13, and S14), with the remaining studies completed in North America (S2, S11, and S12) or Australia (S4 and S8). Participant ages ranged across the lifespan with 5 studies testing interventions in child or adolescents samples (S3, S5, S6, S8, and S10), 3 in adolescent or adults (S2, S4, and S14), and 5 studies focused exclusively on adults (S1, S7, S9, S11, and S12). Some studies were restricted to specific populations (eg, men who have sex with men (S11) or individuals with Autism Spectrum Disorder (ASD), or Attention Deficit Hyperactivity Disorder ([ADHD], S14). The treatment target for the psychological interventions varied and included specific interventions for individuals with depression (S7, S9, S10, and S11), anxiety (S2), substance use (alcohol S1, cannabis S13), or general mental health support (S3, S4, S5, S8, and S12). Intervention length or number of sessions was highly variable, ranging from studies reporting single session interventions (S2, S5, S6, and S8), between 2-5 sessions (S3, S4, and S10), 5-10 sessions (S1, S7, S11, and S13), 10 or more (S14), and 1 intervention lasting 12 months with access to regular text-based counseling (S9). For 1 study, it was difficult to assess the frequency of Web-based text counseling (S12).

In terms of the intervention effectiveness, 1 study was abandoned due to low recruitment rates (S3). Studies that included in their design a waitlist (WL) condition (S1, S7, S10, and S13) showed significant and sustained improvements for participants following the intervention condition compared with WL. S1 text-based chat therapy sessions showed improved outcomes versus WL condition, with significant reduction of large effect in alcohol consumption and improved Quality of Life Scale (QOLS) scores at 3 months and 6 months. For S7, 38.1% (43/113) allocated to text-based therapy intervention compared with 24% (23/97) allocated to WL met study recovery criteria (Beck Depression Inventory [BDI] score<10) at 4 months post-intervention, whereas at 8 months, 42.2% (46/109) versus 25.7% (26/101) met study recovery criteria. In S10, online text-based intervention showed greater improvement at 9 weeks and 4.5 months compared with WL participants. In S13, compared with WL, the selfhelp+chat intervention showed significant posttreatment reduction in cannabis (days of use per week), though groups did not differ on mental health outcome measures (5-item Mental Health Inventory [MHI-5]). Some studies did not include comparison conditions (S4, S11, and S14) and instead, examined change in psychological distress, life satisfaction as a function of the number of sessions completed (S4), or completed pre or post differences on measures of self-esteem (S14) or symptoms of depression or anxiety (S11).

Studies with comparison conditions (S1, S2, S3, S5, S6, S8, and S9) included treatment as usual (TAU) and/or specifically designed comparison conditions (eg, S9). TAU included telephone-based intervention (S5, S6, and S8) and face-to-face intervention (S2, S3, and S12). Compared with TAU, synchronous text-based interventions showed post treatment improvement that was not superior to the TAU condition (S2, S6, and S12). Compared with TAU, one synchronous text-based intervention showed post treatment improvement, though the TAU condition showed marginally greater impact (S8: telephone), and another showed the inverse with larger effect size reported for text-based intervention compared with TAU (telephone: S5).

### Feasibility and Acceptability Studies

Information about each included study S15-S24 [32-41] is provided in Multimedia Appendix 1, and a more detailed description of each study is provided as supplementary information in Multimedia Appendix 2.

The wide range of the included feasibility and acceptability studies show that current research is not limited to evaluating Web-based synchronous text-based intervention as a direct substitute for face-to-face therapy. In fact, most studies made use of at least one additional technical possibility to support or augment interventions. For example, S24 stated that synchronous text-based interactions cover only half of the content of a regular intervention, they also successfully evaluated in their study the utilization of pre-developed responses that could be reused in conversation with different clients.

Other technological solutions that were part of studies included chatbots, dynamic customer personalized learning modules, and progress monitoring through Web-based questionnaires, as well as support for scheduling, customer psycho-education, and training. Interventions were mostly provided by professionals. However, novel approaches in synchronous text-based interventions included trained volunteers delivering basic and specific mental health services (S15-17), as well as fully automated interventions where a computer program was substituted for the therapist (S20). A promising approach was to make better use of the time that participants wait before they engage with a human therapist. For example, S18 and S19 showed that the time participants spend in a virtual waiting room could be used meaningfully for them to complete questionnaires. In the future and similar to the intervention described by Lindenburg and colleagues (see S21), automated concierge services that support participants or clients while they wait to access counseling may involve tools for pre-assessment that could direct clients to psycho-educational information relevant to their needs. The use of Web-based chat services was mentioned to be the main preference for participants (S22) and was shown to have the potential to easily reach a geographically dispersed population in most studies. Web-based chat services may also help individuals to reduce the barrier of help seeking and motivate them to seek face-to-face professional help if required (S22).

## Discussion

### Principal Findings

There is no doubt that individualized chat-based psychological support is a growing area in the mental health care sector, and this review demonstrates a concomitant increase in the number of published studies reporting outcomes from individualized chat-based psychological therapies. Dowling and Rickwood [4] reported 6 in their systematic review, and we describe an additional 8 studies that have been published in the intervening 3 years and 7 reporting additional features of Web-based synchronous text-based interventions. Whereas Dowling and Rickwood [4] in their previous systematic review reported tentative support for Web-based chat counseling with evidence indicating it was as effective as face-to-face support [19,29], the authors noted limitations related to the overall poor quality of studies. Similarly, our findings are mixed, though overall positive. Compared with WL conditions, synchronous text-based interventions showed post treatment improvement, and synchronous text-based interventions appear to be as effective as TAU, though findings were somewhat varied. Mixed findings are likely to be explained, at least in part by the high variability in intervention design. Length (range: single session-1 year) and target (eg, general health and well-being, depression symptomatology, and ASD symptoms), as well as the type of intervention administered (eg, motivational interviewing and cognitive behavioral therapy), varied amongst the studies. For these reasons, though improvement on measures of outcomes was reported for most studies, it is difficult to say at this stage whether one-to-one synchronous text-based communication is preferentially suitable to one type or length of intervention, or mental health condition over others.

In this review, we note that the only trial that compared a synchronous text-based intervention with a face-to-face condition was abandoned because of low recruitment rates [20]. However, several studies reported no immediate advantage of Web-based text-based communication over self-help, enhanced support, or telephone intervention [18,23,26], whereas others showed significant benefits to Web-based, text-based communication above telephone delivered support [22]. All reported that Web-based support was significantly beneficial in comparison with WL [18,24,27,30], though others [26,29] reported no advantage of Web-based chat support interventions compared with TAU conditions.

Studies indicate that Web-based chat is acceptable and feasible as a mode of therapeutic support, with participants in some studies indicating a preference for this mode of interaction over telephone call. Interestingly, a study by Fukkink et al [23], which also reported that children rated the quality of chat conversations equally or higher than a telephone conversation, found that the average text-based session time was 24 min, almost three times as long as the telephone session (9.3 min). King and colleagues [25] also observed longer counseling times for their text-based intervention, with typical session times between 50-80 min for text-based counseling versus 45-60 min for telephone counseling. Despite this, counseling, satisfaction outcomes in this study were higher in the telephone condition, notwithstanding longer session time in the text-based condition. Increased session length and lower satisfaction may be a consequence of reduced availability of conversational cues during text-based communication, such as the absence of intonation and other nonverbal communication. Nevertheless, the extended times for Web-based chat interventions could also simply be a result of the slower communication channel (it generally takes longer to type than say something). This was shown by Rodda et al [41], who indicated that text-based exchanges generated approximately half the words of an oral conversation. Additionally Fukkink et al [23] found even stronger differences, with an average of 32 words a minute (chat) versus 143 words a minute in the telephone conversation. When taken together, if this mode of intervention delivery generates similar effectiveness but longer session times, generating fewer words, and in some groups, lower satisfaction, this draws into question the clinical practicality of this mode of delivery. Future studies will need to examine how and whether synchronous text-based interventions can best be utilized in the mental health sector and what additional innovations, or constraints may be required before this mode of intervention delivery is financially and logistically viable.

The studies described here have several limitations. Sample sizes were often too small to provide adequate power to studies to explore potential mediating and moderating effects such as other participant or intervention characteristics that may have impacted the outcomes. The ability to detect potential subgroups for whom the intervention may have been particularly effective was also hampered by small sample sizes. Attrition rates remain problematic as well as potential self-selection bias of samples and inadequate (or absent) comparison conditions. However, it should be noted that Crutzen, and colleagues [20] abandoned and then reported their study for this very reason. The lack of adequate documentation or statistical control of additional treatments participants sought out during the interventions is also of concern. The study by Kordy et al [26] is interesting for this reason. They report comparisons to a Web-based chat condition with a quite substantial TAU condition, as well as a structured intervention without a chat component. The heterogeneity of samples participating in studies is also a limitation that requires consideration. Whereas several studies state specific psychiatric or substance dependency criteria for inclusion, others recruited cross diagnostically and lacked adequate assessment of comorbid psychiatric conditions. Furthermore, self-report and Web-based completion of psychometric measures, as well as when and where measures were completed, was noted by Rodda and Lubman [41] as potential limitations of Web-based chat-based interventions that may influence the reliability and validity of study findings. Finally, the “dose” and intensity of Web-based chat interventions was poorly articulated by many studies and varied between the studies with interventions ranging from a single session to 12 months. Follow-up time frames were also absent or inadequate for some studies that in turn limited the ability to examine the durability of any treatment outcome gains, as well as the generalizability of positive outcomes.

### Limitations

It is important to note that some of the journals reporting synchronous text-based individualized interventions were not yet registered with the major databases. Therefore, they were not identified as part of the initial database search. Also, many innovations may have been published in technology conferences. Though the primary databases for these conference manuscripts were included (ie, ACM; IEEE), our search may not have captured other technically oriented manuscripts. The fact that these innovations do not appear in our review, even though the major engineering databases were searched, is likely because they lack the type of evaluations required in the mental health domain. The type of outcome measures of technologically driven papers is usually related to the accuracy of the algorithms and not mental health. More collaboration between computing scientists and mental health researchers is required to address this issue.

Another limitation impacting this area of research and that impacted our search yield in the current review relates to the lack of standard nomenclature to describe Web-based psychological interventions. This issue has been discussed in previous systematic reviews [1,4]. For example, when refining our database abstract or title or keywords search strategy, we found that including the keyword “support” in our search query would have resulted in 6759 instead of 591 returns in MEDLINE (on November 4, 2016). Thus, given the heterogeneity in terminology applied in this relatively new field of research, we suspect that despite our best effort, due to terminology used by some studies, these manuscripts may have been missed in our literature search. In order to minimize the likelihood of missing relevant research, we also reviewed reference lists of included manuscripts (ie, backward and forward reference search).

### Future Directions

The authors expect a strong trend toward more Web-based mental health services delivered using synchronous text-based systems. This trend is already visible with the recent introduction of commercial platforms such as Koko (http://www.itskoko.com) or Ginger.io (http://Ginger.io) that for a fee provide psychological support and include individualized chat-based access to clinicians and in the case of Koko, moderated peer support for mental health problems. Other platforms are also beginning to offer infrastructure for chat-based services (eg, qntfy.com). As aforementioned, new services from the private sector populating the mental health space are largely unevaluated, and therefore, there is limited research evidence regarding how effectively these services support mental health and well-being. Despite the promise of this mode of intervention and commercial interest in its application, the evidence described here would seem to suggest that the clinical viability of this mode of delivery has yet to be proven.

The introduction of conversational agents might play an important role. In the study described by Gaffney et al [37], a conversational agent was trained to utilize principles of Method of Levels (MOL) therapy. The optimization and augmentation of human service through artificial intelligence is speculated to be a promising area of future research and commercial application [42,43]. Another example, 7Cups (www.7cups.com) has introduced a “therapist bot” that greets users and carries out basic assessments to aid in evaluating, based on algorithms, their needs and how best to connect them with a human therapist to chat with on the Web. However, future research still needs to address the suitability of this type of technology for mental health and well-being interventions. For instance, it remains unclear whether it is more suited to particular mental health problems, is best suited to short- or long-term intervention, or whether it is best applied as an adjunct or screening tool to streamline services.

Feasibility studies indicate that text-based interventions are well tolerated by users seeking help for mental health concerns. A significant advantage of this mode of intervention noted by studies was the availability and immediacy of support. Users may prefer immediate support when they seek it, even when trained volunteers or artificial agents are delivering this support. For these reasons, overall current research suggests that synchronous text-based communication between clients and intervention providers is feasible and acceptable for a wide range of users. However, as to whether these interventions are an adequate substitute for non-text based intervention cannot be answered based on the current level of evidence from studies of efficacy.

### Conclusions

This review provides an evaluation of individual synchronous Web-based chat technologies as a mode of psychological intervention and support. Based on the current evidence of the application of this technology in this area of mental health research, we see tentative support for this mode of intervention. Interventions utilizing text-based synchronous communication showed better outcomes compared with WL conditions and overall equivalent (though variable) outcomes compared with TAU, and were at least as good as the comparison interventions. However, the issue of whether these technologies are cost effective in clinical practice remains a consideration for future research studies. New technologies are rapidly changing the area by increasing the capacity to automate some of the components of synchronous text-based intervention; however, our conclusions remain tentative because of the limited number of studies reporting randomized controlled trial (RCT) evaluation of interventions and adequate follow-up procedures. This review raises the need for more interdisciplinary work in evaluating new Web-based synchronous text-based technologies.

## Appendix

Multimedia Appendix 1 Summary of feasibility studies.

Multimedia Appendix 2 Narrative summary of feasibility studies.

Multimedia Appendix 3 Summary of efficacy studies.

## Abbreviations

TAU: treatment as usual
SMS: short message service
NLP: natural language processing
MMS: multimedia messaging service
MEDLINE: Medical Literature Analysis and Retrieval System Online
DSM: Diagnostic and Statistical Manual of Mental Disorders
ADHD: attention-deficit/hyperactivity disorder.
ASD: autism spectrum disorder.
QOLS: Quality of Life Scale.
BDI: Beck Depression Inventory.
MHI-5: 5-item Mental Health Inventory.
