# Supplementary material for: Application of Synchronous Text-Based Dialogue Systems in Mental Health Interventions: Systematic Review
Source: J Med Internet Res. 2017 Jul 21;19(8):e267. doi: 10.2196/jmir.7023 (PMC5595406; doi:10.2196/jmir.7023)
Supplement: Multimedia Appendix 1 [file jmir_v19i8e267_app1.pdf]

Multimedia Appendix 2. Summary of feasibility and acceptability studies of synchronous text-based psychological interventions.

Study	Type of intervention	Other technologies	Support service	Interventionists	Population	Outcome	Implications
S15. Baumel (2015), United States [32]	Web and app based chat system	Web-based training modules (text, video, and quiz); Online profiles; Automatically triggered counseling tips for listeners during chat; Web-based questionnaire	Emotional support, for example, relationship issues, loneliness, depression, anxiety, and other emotional problems	Volunteers who conducted a computerized training program on active-listening	General population	Users rated service provide by volunteers as helpful as psychotherapy and as more genuine	Training of volunteers can be made scalable with Web-based computerized modules on general counseling skills
S16. Baumel et al (2016), United States [33]	Web and app based chat system	Web-based training modules (text, video, and quiz) Online profiles; Disorder specific training modules; Web-based questionnaire	Emotional support to complement ongoing treatment	Trained and experienced volunteers who conducted Web-based training in active listening and a disorder specific training	Schizophrenia-spectrum disorder patients	Users rated service provided by volunteers as helpful as psychotherapy and more genuine.	Volunteers can be trained on the Web with computerized modules to provide emotional support for specific disorders
S17. Baumel et al (2016), United States [34]	Web and app based chat system	Web-based training modules (text, video, and quiz) Online profiles; Disorder specific	Single session of chat support	Trained and experienced volunteers who conducted Web-based training in active listening and a disorder specific training	Women with perinatal depression and anxiety	Service was rated as usable and useful. Strong interest for peer-support	Online support outside regular therapy times could help to meet patients' support needs

Study	Type of intervention	Other technologies	Support service	Interventionists	Population	Outcome	Implications
		training modules					
S18. Dowling and Rickwood (2014), Australia [35]	Web-based chat service (eheadspace)—multiple sessions	Virtual waiting room<br>Web-based advertisement<br>Email<br>Web-based questionnaires (pre and post intervention)	Support relating to psychological, psychosocial, vocational, and educational issues	Youth mental health clinicians with qualifications in psychology, occupational therapy, mental health nursing, or social work	Young people 16-25 years with mental health issues (eg, anxiety and depression, relationships; suicidal ideation and self-harm)	Highly distressed visitors but significant reduction after intervention	Although multi-session interventions have better treatment outcomes, a single session is the preferred choice of visitors
S19. Dowling and Rickwood (2016), Australia [36]	Web-based chat service (eheadspace)	Virtual waiting room<br>Web-based advertisement<br>Web-based Questionnaires	Support relating to psychological, psychosocial, vocational, and educational issues	Youth mental health clinicians with qualifications in psychology, occupational therapy, mental health nursing, or social work	Young people 16-25 years in virtual waiting room of mental health chat-service	Web-based chat clients: are highly distressed; have very low levels of hope; have high treatment outcome expectations	Uses Web-based tools such as questionnaires beforehand. The data collected with these tools could help session preparation
S20. Gaffney et al (2014), United Kingdom [37]	Automated instant messenger chat interface(s) with a computerized therapist	Manage Your Life Online chatbot; ELIZA, a chatbot from 1966	Supporting the process of recognition using Method of Levels psychotherapy approach	Chatbot(s)	Preselected university students with a problem that causes them distress	Distress, depression, anxiety, and stress were reduced after interactions with one of the chatbots	Chatbots could potentially take over some restricted and predefined tasks in an online psychotherapy session
S21. Lindenberg et al. (2011), Ireland [38]	Web-based chat service with counselor and group	Psychoeducational Web page,<br>Online forum, monitoring and feedback program	Prevention of eating disorders	Professional counselors	College students	Chat features were considered a good concept; Few participants actually used individual	Web-based stepped-care modules can be used to augment and personalize interventions

Study	Type of intervention	Other technologies	Support service	Interventionists	Population	Outcome	Implications
						chat; Most participants found program through Web-based search (67.65%)	
S22. Pietrabissa, et al (2015), Italy [39]	Chat functionality in Facebook	Advertisement through public Facebook psychology issue discussion groups; Email; Friendship request on Facebook; Google forms for questionnaire	Preliminary advice and discussion around participants' main psychological issues (eg, depression, anxiety, and relationship difficulties	Professional psychologist	All age groups, 44.8% with at least one session with a psychologist in the past	Web-based articles on the Web page and social network services, as well as Web -searches were the main pathway to the service. Most (79.2%) of participants stated to prefer the Web over in-person consultation; Mostly because of immediacy of support, economic convenience and lack of knowledge about other services. About half (48%) of the respondents to the follow-up questionnaire requested further	Social network sites such as Facebook have become increasingly popular and should be considered as a platform for psychological service for individuals (with Facebook chat), as well as a means to communicate and inform larger amounts of people (in Facebook groups); Web-based chat can be a gateway and help motivate people seeking professional help

Study	Type of intervention	Other technologies	Support service	Interventionists	Population	Outcome	Implications
						psychological support	
S23. Reynolds et al (2013), United States [40]	Chat (or email exchange)	Web-based questionnaire Web-based site with personal login for clients and 2 weeks history; Reminder email service	Psychotherapy (including depression and anxiety)	Predominantly US licensed psychotherapists	19-55 year old people recruited from various mental health sites (Web-based and regular)	Session impact and therapeutic alliance scores were equally as strong or stronger than for face-to-face therapies in other studies; Lower arousal ratings for intervention compared with previous face-to-face data	Web-based text based psychotherapy has promising potential as a therapy form; Online interventions might benefit from a calming effect compared with face-to-face
S24. Rodda and Lubman (2014), Australia [41]	Web-based chat	Email<br>Push-page technology (to reuse previously developed content in chat)<br>Profile page for visitor with referral info, assessment results, and previous sessions	Counseling service for problem gambling	Trained gambling counselors	Open to all age groups	About one third of registered visitors returned for second session; New treatment seekers are more often male and under 30 years	Web-based chat is a viable alternate mode of service delivery that attracts new treatment cohorts

References

32. Baumel A. Online emotional support delivered by trained volunteers: users' satisfaction and their perception of the service compared to psychotherapy. J Ment Health 2015; 24(5):313-20

33. Baumel A, Correll CU, Birnbaum M. Adaptation of a peer based online emotional support program as an adjunct to treatment for people with schizophrenia-spectrum disorders. Internet Interv 2016 May; 4(Part 1):35-42

34. Baumel A, Schueller SM. Adjusting an available online peer support platform in a program to supplement the treatment of perinatal depression and anxiety. JMIR Ment Health 2016 Mar 21; 3(1):e11

35. Dowling M, Rickwood D. Investigating individual online synchronous chat counselling processes and treatment outcomes for young people. Advances in Mental Health 2014; 12(3):216-224

36. Dowling M, Rickwood D. Exploring hope and expectations in the youth mental health online counselling environment. Comput Human Behav 2016 Feb; 55(Part A):62-68

37. Gaffney H, Mansell W, Edwards R, Wright J. Manage Your Life Online (MYLO): a pilot trial of a conversational computer-based intervention for problem solving in a student sample. Behav Cogn Psychother 2014 Nov; 42(6):731-46

38. Lindenberg K, Moessner M, Harney J, McLaughlin O, Bauer S. E-health for individualized prevention of eating disorders. Clin Pract Epidemiol Ment Health 2011; 7:74-83

39. Pietrabissa G, Manzoni G, Algeri D, Mazzucchelli L, Carella A, Pagnini F, Castelnuovo G. Facebook use as access facilitator for consulting psychology. Aust Psychol 2015 Jul 20; 50(4):299-303

40. Reynolds Jr DJ, Stiles WB, Bailer AJ, Hughes MR. Impact of exchanges and client-therapist alliance in online-text psychotherapy. Cyberpsychol Behav Soc Netw 2013 May; 16(5):370-7

41. Rodda S, Lubman DI. Characteristics of gamblers using a national online counselling service for problem gambling. J Gambl Stud 2014 Jun; 30(2):277-89
