# Supplementary material for: Application of Synchronous Text-Based Dialogue Systems in Mental Health Interventions: Systematic Review
Source: J Med Internet Res. 2017 Jul 21;19(8):e267. doi: 10.2196/jmir.7023 (PMC5595406; doi:10.2196/jmir.7023)
Supplement: Multimedia Appendix 2 [file jmir_v19i8e267_app2.pdf]

Appendix A: Feasibility studies narrative summary

S15 – S17. Baumel et al. (2015-16), United States [32-34]

Baumel et al. evaluated various adaptations of 7 Cups of Tea (7Cups), a chat platform for online mental health support. The 7Cups platform trains volunteers to deliver online emotional support and provides the online infrastructure to deliver emotion support to interested individuals. Initial evaluation of a convenience sample of 865 users indicated high user satisfaction with the support provided by 7Cups volunteers [32], and of those users who had received psychotherapy previously 7Cups was rated as useful as previous therapy, though the two therapies were considered to hold different benefits. Baumel and colleagues [33] extended this work to adapt 7Cups to provide support to people with schizophrenia-spectrum disorders. A clinician-training program (called SUPPORT) was developed and provided to volunteers (n=168) via the platform. The authors report that additional training was found to increase volunteer's knowledge and confidence. Further, a small sample of individuals (n=10) with a diagnosis of schizophrenia (or related disorder) was recruited to the study to rate the usefulness and helpfulness of the platform. They reported that the platform would be a usable and helpful resource for emotional and social support. The platform has been similarly adapted for use by populations with perinatal depression or anxiety and although only a small sample if users were recruited to the study (n=9), the authors report similarly positive evaluations of 7Cups by users [34].

S18. Dowling and Rickwood (2014), Australia [35]

This study evaluated the effects of synchronous online chats counselling conducted with young people between 16 and 25 years old. A total of 49 chat transcripts as well as psychological pre and post-treatment scores were assessed. Counselling sessions lasted for approximately 50 minutes each. Thirty-three transcripts were from single sessions whereas 16 transcripts were combined from two sessions. Participants were recruited in the virtual waiting room of the service using an online advertisement. Pre-session data was collected before participants received online counselling whereas invitations to fill-out post-session surveys were sent to the participants via email. Attrition numbers were not reported. Overall the level of psychological distress (K10) decreased significantly between the two observations from 34.92 (SD = 7.66) at pre-test to 31.65 (SD = 8.75) at post-test. Children's Hope Scale and Satisfaction with Life Scale were also applied however no significant effects were found between the two time-points. Additionally, counselling progress and depth rating

were quantified from the chat transcripts using the Counselling Progress and Depth Rating Instrument. Session progress and session depth score correlated positively (r=0.29* and r=0.3*) with psychological distress however no significant correlation was found with life satisfaction or hope.

S19. Dowling and Rickwood (2016), Australia [36]

This study was conducted with 16 to 25-year-old visitors of eheadspace, an Australian chat-counselling service for young people. Participants were recruited from February to 2013 to November 2013 with a written advertisement on the webpage with the question: "Are you aged 16-25 and would like to participate in a survey about your experience?" From the 1407 participants who started the questionnaire 1033 completed it. Participants were predominantly female (86.4%), under 18 years old (57%) and lived in urban locations (66%). Participants' rating of the Children's Hope Scale and the Brief Multidimensional Student's Life Satisfaction Scale were low, whereas the ratings in the Treatment Outcome Expectations Scale and the Kessler Psychological Distress Scale (K-10) were high.

S22. Gaffney et al (2014), United Kingdom [37]

This study compared a self-help computer software conversation system "Manage Your Life Online" (MYLO) with the natural language program called ELIZA which was released in 1966. The version of MYLO used in the study was developed to identify 52 themes and to provide relevant terms and questions for each theme. A total of 48 students from the University of Manchester took part in the study and were randomly assigned by coin toss to either of the two conditions. Each session took 45 minutes of which up to 20 minutes were dedicated to the conversation with the computer program. Four participants in the MYLO condition were excluded due to server malfunction and two participants in the ELIZA condition due to incomplete measures. The results of the study showed that both computer programs had a significant impact on reducing the distress level that the problem they discussed was causing and the rating on the Depression, Anxiety and Stress Scale; the differences between the two conditions were not significant. Ratings of helpfulness and problem resolution however were significantly higher for MYLO.

S21. Lindenberg et al. (2011), Ireland [38]

This paper presents a study called “Appetite for Life” aimed to prevent eating disorders. Part of the intervention was a 30-minute one-to-one chat with an online counsellor, several times a week as well as a counsellor lead group chat once a month. The main parts of the intervention were however, educational material, supportive monitoring, a feedback system and a moderated forum. Participants that scored moderately to high on a Weight Concern Scale and/or reported non-severe symptoms on the Short Evaluation of Eating Disorders questionnaire and did not currently undergo treatment.

In total 457 participants were recruited among the students of the Trinity College Dublin, Ireland and screened for eligibility. Two participants, who fully activated their account, but had “no risk and no symptoms” did only get access to the forum but not the rest of the program whereas 78 eligible users registered and had access to the full program. However only 2 of the 34 users who returned the questionnaire reported of having used the chat service during the two months of their participation. Nevertheless, 28 out of 33 users rated the *individual consultation chat* as a good concept and 17 out of 31 rated the *group consultation chat* as a good concept. The rating of the perceived helpfulness of both the individual and group chat feature was however low with 4 out of 23 participants rating the individual and 2 out of 23 rating the group chat as *helpful* on a scale that included (“helpful”, “neutral”, “harmful” and “don’t know”).

S22. Pietrabissa, et al (2015), Italy [39]

The study used Facebook chat to examine the reasons why individuals seek online psychological consultations. The availability of a 30-min Facebook chat consultation was advertised. A total number of 284 participants participated in a session with a professional psychologist, of which 259 completed the initial survey, which included questions on their presenting problems and the reasons that led them to the online consultation. During the 30-min consultation, the psychologist provided relevant information and motivated the participant to seek follow-up face-to-face advice. After the 30-min session, participants were asked to fill out a second questionnaire evaluating their degree of satisfaction with the service. A third questionnaire was sent out to participants a month later to determine whether an in-person consultation had been requested. 79.2% of the first survey participants stated that their preference was to attend an online rather than an in-person consultation, mainly

because of the need for immediate help, followed by the economic convenience of the service. Depression was the greatest problem reported by 82 participants (31.8%), followed by 62 (24%) participants who reported anxiety problems and 47 (18.2%) with relationship difficulties. A total of 174 participants completed the second questionnaire, reporting on average that they were highly satisfied with the S.I.P.O online consultation and had a high motivation to seek further professional psychological support. Following their online consultation, 26 out of 54 individuals who completed the third questionnaire requested further psychological support. The ease of access to a free of charge chat consultation could have been an important factor supporting the efficiency of FB in reaching people with psychological care needs. However, as 51.9% of the 54 users who answered the third survey had not turned to a professional psychologist, the fact that the FB service was free of charge could have reduced participant's perception of the treatment's value.

S23. Reynolds et al (2013), United States [40]

This study evaluated an unspecified form of online intervention that was either email or chat based. Thirty therapists and thirty clients were recruited and had at least 6 weeks of exchange. The ratings of the Session Evaluation Questionnaire (SEQ) and Alliance (ARM-12) where compared with previously collected data from face-2-face interventions and were overall comparable with the expectations of Text therapist SEQ scores which were greater.

S24. Rodda and Lubman (2014), Australia [41]

The study evaluated a real-time chat based counselling services for problem gambling in Australia. It assessed the characteristics of users and the uptake of the service. In a period of two years 1,722 unique users chatted with a trained gambling counselors about their own gambling problems. Their average session typically lasted 45 minutes and covered about half the content that would be covered in face-2-face or telephone sessions. The authors observed that about one-third of registered users returned to the service for more counseling sessions; the actual number of returning users however, was estimated to be much higher because the service can also be used anonymously without registration or login. Specific to this chat-based intervention is the use of a push-page technology to introduce canned content to the chat which then is discussed during the session.

References

32. Baumel A. Online emotional support delivered by trained volunteers: users' satisfaction and their perception of the service compared to psychotherapy. J Ment Health 2015; 24(5):313-20

33. Baumel A, Correll CU, Birnbaum M. Adaptation of a peer based online emotional support program as an adjunct to treatment for people with schizophrenia-spectrum disorders. Internet Interv 2016 May; 4(Part 1):35-42

34. Baumel A, Schueller SM. Adjusting an available online peer support platform in a program to supplement the treatment of perinatal depression and anxiety. JMIR Ment Health 2016 Mar 21; 3(1):e11

35. Dowling M, Rickwood D. Investigating individual online synchronous chat counselling processes and treatment outcomes for young people. Advances in Mental Health 2014; 12(3):216-224

36. Dowling M, Rickwood D. Exploring hope and expectations in the youth mental health online counselling environment. Comput Human Behav 2016 Feb; 55(Part A):62-68

37. Gaffney H, Mansell W, Edwards R, Wright J. Manage Your Life Online (MYLO): a pilot trial of a conversational computer-based intervention for problem solving in a student sample. Behav Cogn Psychother 2014 Nov; 42(6):731-46

38. Lindenberg K, Moessner M, Harney J, McLaughlin O, Bauer S. E-health for individualized prevention of eating disorders. Clin Pract Epidemiol Ment Health 2011; 7:74-83

39. Pietrabissa G, Manzoni G, Algeri D, Mazzucchelli L, Carella A, Pagnini F, Castelnuovo G. Facebook use as access facilitator for consulting psychology. Aust Psychol 2015 Jul 20; 50(4):299-303

40. Reynolds Jr DJ, Stiles WB, Bailer AJ, Hughes MR. Impact of exchanges and client-therapist alliance in online-text psychotherapy. Cyberpsychol Behav Soc Netw 2013 May; 16(5):370-7

41. Rodda S, Lubman DI. Characteristics of gamblers using a national online counselling service for problem gambling. J Gambl Stud 2014 Jun; 30(2):277-89
