# Supplementary material for: Application of Synchronous Text-Based Dialogue Systems in Mental Health Interventions: Systematic Review
Source: J Med Internet Res. 2017 Jul 21;19(8):e267. doi: 10.2196/jmir.7023 (PMC5595406; doi:10.2196/jmir.7023)
Supplement: Multimedia Appendix 3 [file jmir_v19i8e267_app3.pdf]

Multimedia Appendix 1. Summary of efficacy studies of synchronous text-based psychological interventions.

**Study**	Population or sample	Design (eg, RCT[a])	Eligibility criteria (specific diagnosis)	Intervention and duration (eg, single session, 6 week program)	Control condition (eg, TAU[b])	Attrition	Main findings
S1. Blankers et al (2011), Netherlands [18]	N=205 adults with drinking problems<br><br>Therapy alcohol online n=68, Age: mean=41.9; 49% male<br><br>Internet-based self-help n=68, Age: mean=41.1, 49% male<br><br>Waitlist n=69, Age mean=43.7, 51% male	RCT	18 years or older; AUDIT[c] score>8 to report drinking an average of more than 14 standard drinks a week	Synchronous Web-based therapy based on CBT[d] and motivation interviewing conducted in up to 7 text-based chat-therapy sessions (TAO[f])<br><br>Assessments at baseline, 3 and 6 months	Self-help Alcohol Online, a fully automated treatment intervention based on CBT and motivational interviewing (SAO[g])<br><br>Untreated waiting-list control group (WL[e])	TAO=36% at 3 months did not complete assessments, 40% at 6 months did not complete assessments<br><br>SAO=30% at 3 months did not complete assessments; 43% at 6 months did not complete assessments<br><br>WL= 26% at 3 months did not complete assessments, and 38% at 6 months did not complete assessments	Alcohol consumption: significant effects for TAO versus WL (*P*=.002) and for SAO versus WL (*P*=.03) on alcohol consumption at 3 months<br><br>Differences between TAO and SAO were not significant at 3 months (*P*=.11) but were significant at 6 months (*P*=.03), with larger effects obtained for TAO<br><br>QOLS[h] score: differences between TAO and WL were significant at 3 months (*P*=.11) with

							larger effects obtained for TAO<br><br>Differences between TAO and SAO were not significant at 3 months (*P*=.37) but were at 6 months (*P*=.02) with larger effects obtained for TAO
S2. Cohen and Kerr (1999), United States [19]	N=24 Adolescents and adults (self-identified as wanting help for dealing with anxiety)	RCT	Help-seeking for anxiety	Single session<br><br>Sessions conducted by psychology graduate students	TAU (face-to-face)		Anxiety: STAI[i] scores reduced significantly for both groups (chat and face-to-face)<br><br>No significant differences between intervention groups
S3. Crutzen et al (2014), Netherlands [20]	N=12 Adolescents with psychosocial problems<br><br>Web-based chat (n=5)<br><br>Face-to-face (n=7)	RCT	SDQ[j] total score of 12 or higher on the SDQ	Three Web-based chat consultations with a YHC[k] worker (over a 3 month period)	Three face-to-face consultations with a YHC worker (over a 3 month period)	N=360 were invited to join the study; N=70 met secondary exclusion criteria<br><br>n=165 declined to participate; n=74 not invited to participate; n=12 wrongly excluded;	Trial abandoned due to low recruitment rates

						n=10 no information;<br>n=17 drop out before participation	
S4. Dowling and Rickwood (2015), Australia [21]	N=152 adolescents and adults who accessed a mental health website<br><br>Age range: 16-25 years, Mean=17 (SD[l] 2.45); 12.2% male	Naturalistic study with pre or post measures	The age range of 16-25 years	Web-based support provided by fully qualified and supervised mental health clinicians, for example, psychologists, mental health nurses, occupational therapists, and social workers		N=506 completed baseline survey<br><br>n=354 (70%, 354/506) did not complete a therapy session<br><br>n=152 accessed Web-based chat website and responded to follow-up<br><br>Of these, n=38 (25%, 38/152) did not progress to have a Web-based session;<br>n=62 (40.8%, 62/152) had one session;<br>n= 52 (34.2%, 52/152) had 2-5 sessions	No differences between participants at baseline<br><br>Psychological distress: all participants improved significantly with moderate to large ES[m] for time (*P*<.001, partial eta^2=.118) regardless of amount of counseling received<br><br>Life satisfaction: a significant though small ES for time, with an increase in life satisfaction for all groups. (*P*=.009, partial eta^2=.046). No differences were found according to the amount of

							Web-based counseling received<br><br>Hope: no main effects but participants with 1 (*P*=.019, d=.25) or more (*P*=.008, d=.36) session increased significantly with a small to medium ES
S5. Fukkink and Hermanns (2009), Netherlands [22]	N=12,873 children who contacted helpline and answered 2 questions before intervention<br><br>n=902 children and adolescents with first-post intervention questionnaire completed<br><br>Age range 8-18 years<br><br>Web-based chat: n=339 Age: mean=13.8; 11.5% male	Naturalistic comparison	Aged less than 18 years	Single session Web-based chat<br><br>Problem-solving based counseling session	TAU (telephone conversation)	11,971 did not complete postintervention questions<br><br>At 1 month, 24.7% (223/902) available for follow-up: n=119 Web-based chat; n=94 telephone	Well-being increased for participants in both groups following intervention (*P*<.001): Web-based chat (ES=0.62, medium) and telephone (ES=0.34, small)<br><br>Perceived burden of problem decreased in both groups following intervention (*P*<.001): Web-based chat (ES=0.44,

	Telephone n=563 Age: mean=12; 21.3% male						medium) and telephone (ES=0.12, small)<br><br>Follow-up analysis at 1 month, showed that changes in well-being and perceived burden of the problem were stable for both intervention conditions<br><br>Intervention comparison showed that Web-based chat was slightly more effective in both improving children's sense of well-being and decreasing the perceived burden of the problem<br><br>Overall, children were satisfied with the support they received from both chat and telephone interventions

S6. Fukkink and Hermanns (2009), Netherlands [23]	N=110 Children and adolescents who contacted the helpline<br><br>Age range 9-17 years<br><br>Web-based chat n=53; 16% male<br><br>Telephone n=42; 23% male	Selection of conversations from database either phone or chat based	Aged less than 18 years	Single session Web-based chat<br><br>Problem-solving based counseling session	TAU (telephone conversation)	n=15 excluded conversations due to poor quality, or abrupt end to session	From selected conversations, children's well-being increased for both Web-based chat (ES=0.45, medium) and telephone interventions (ES=0.40, medium)<br><br>Perceived burden of the problem decreased for online chat (ES=0.36, small) and telephone (ES=0.20, small)<br><br>There were no significant differences between chat and telephone interventions
S7. Kessler et al (2009), United Kingdom [24]	N=297 adults with depression<br><br>Age range 18-75 years<br><br>Intervention n=149	RCT	New episode of depression (diagnosed within preceding 4 weeks) and BDI score >14<br><br>No treatment for depression within	16 weeks duration<br><br>CBT-based treatment with online therapist<br><br>5-10 sessions	WL	Online CBT : At 4 months' follow-up, 92 (62%, 92/149) had completed the therapy as intended	At 4 months: intervention BDI mean 14.5 (SD 11.2), (ES=0.81, large)<br><br>Study recovery criteria: n=43,

	Age: mean=35.6; 30% males<br><br>WL control n=148<br>Age mean=34.3; 33% males<br><br>Web-based chat; BDI[n] mean=32.8, SD=8.3<br><br>Waitlist control; BDI mean=33.5, SD=9.3		the preceding 3 months<br><br>No history of bipolar disorder, psychotic disorder, alcohol, or substance misuse	55 min per session		Data was collected for n=113 (76%, 113/149) of the intervention group<br><br>At 8 months' follow-up, n=99 (66%, 99/149) had completed the therapy as intended<br><br>Only 19 (13%, 19/149) received no sessions of therapy, nearly half n=70/149 had had at least eight sessions<br><br>Data was collected for 109 (73%, 109/149) of the intervention group	(38%) reported a BDI of <10;<br><br>WL BDI mean 22.0 (SD 13.5), and n=23 (24%) met BDI recovery criteria<br><br>At 8 months: intervention BDI mean 14.7 (SD 11.6), (ES=0.70, large), Study recovery criteria met by: n=46 (42%)<br><br>WL BDI mean 22.2 (SD 15.2); n=26 (26%) met BDI recovery criteria
S8. King et al (2006), Australia [25]	N=186 children seeking counseling<br><br>Web-based chat n=86; Age: mean=15.4; 5% male	Naturalistic comparison		Single session; Counseling focused on information gathering and problem solving. Duration: 50-80 min.	TAU—telephone duration: 45-60 min		Distress: GHQ-12[o] significantly reduced in both conditions (partial eta squared=0.50, large)<br><br>Session impact: Higher

	Telephone n=100<br>Age: mean=13.1; 33% male						SIS[p] for TAU (telephone) compared with Web-based chat (partial eta squared=0.15)
S9. Kordy et al (2016), Germany [26]	N=232<br><br>SUMMIT n=77<br><br>SUMMIT-PERSON n=79<br><br>TAU n=80	RCT	DSM-IV[q] criteria for MDD[r] with 3 or more major depressive episodes	Over 12 months, participants in both intervention arms received, in addition to TAU, the SUMMIT intervention which comprised intense monitoring via email or a mobile phone, including signaling of upcoming crises, assistance with personal crisis management, and facilitation of early intervention<br><br>SUMMIT-PERSON additionally offered access to regular expert chats	TAU	n=8 (3.4%) participants discontinued participation: (TAU: n=5; SUMMIT: n=3)<br><br>n=17 no interview data<br><br>n=1 (TAU) was interviewed only once because his index treatment lasted 5.5 months<br><br>Lost to follow-up:<br>6 months: n=20;<br>12 months: n=10;<br>18 months: n=12;<br>24 months: n=0	SUMMIT compared with TAU reduced the time with an unwell status (OR[s] 0.48; 95% CI 0.23-0.98) through faster transitions from unwell to well<br>(OR 1.44; 95% CI 0.83-2.50) and slower transitions from well to unwell (OR 0.69; 95% CI 0.44-1.09)<br><br>SUMMIT-PERSON was not superior to either SUMMIT<br>(OR 0.77; 95% CI 0.38-1.56) or TAU (OR 0.62; 95% CI 0.31–1.24)<br><br>The efficacy of

							SUMMIT was strongest 8 months after the intervention<br><br>The accumulated gains are illustrated by the median proportions of well weeks: 52% in SUMMIT and 48% in SUMMIT-PERSON versus 31% in TAU alone
S10. Kramer et al (2014), Netherlands [27]	N=263 adolescents with depressive symptoms<br><br>Intervention (n=131)<br><br>WL (n=132)	RCT	12-22 years of age; a CES-D[t] score of 22 or higher (the cut-off to detect possible cases of depression among adolescents)	Generally limited to 5 chat sessions of Solution-Focused Brief Therapy called PratenOnline	WL	N=592 Excluded: n=329: CES-D<22; no consent signed n=265; baseline not completed n=39<br><br>9 weeks: n=111 (42%); n=56 (praten); n=55 (WL)<br><br>4.5months: n=131 (50%); n=58 (praten); n=76 (WL)	PratenOnline showed significantly greater improvement in CES-D scores than the WL at both 9 weeks and 4.5 months with a small ES at 9 weeks (d=0.18, 95% CI −0.10 to 0.47) and a large ES at 4.5 months (d=0.79, 95% CI 0.45-1.08)
S11. Lelutiu-Weinberger et	N=41 males who have had	Naturalistic study	Male, aged between 18-29	Weekly sessions up to		N=146 eligibility	No significant change in

al (2015), United States [28]	sex with men<br>Age range: 18-29 years<br><br>MiCHAT: n=31<br>Age: mean 25 (SD 3.2)<br><br>Non attendance: n=10		years, negative or unknown HIV[u] status, had used drugs >=5 occasions in the last 90 days, at least one incidence of condomless anal sex in last 90 days	8 1-h motivational interviewing and cognitive behavioral skills-based Web-based live chat sessions		screenings<br><br>Eligible: n=41<br>Intervention: Attended >=1 session n=31<br><br>Completed follow-up n=22<br><br>No session attended n=10; Completed follow-up n=5	depression or anxiety symptom ratings:<br><br>Brief Symptom Inventory Scale: Depressive symptoms Baseline mean 14.48 (SD 6.37) Follow-up: mean 13.37 (SD 6.00) (*P*=.19) D=0.10<br><br>Anxious symptoms Baseline mean 13.29 (SD 6.59) Follow-up: mean 13.1 (SD 6.83) (*P*=.88) D=0.03
S12. Murphy et al (2009), Canada [29]	N=127 adults who contacted an employee and family assistance program provider via phone<br><br>Web-based chat n=26<br>Age: mean=42; 27% male	Naturalistic comparison		Therapy Online counselors gave online counseling<br><br>Intervention unclear<br><br>Counseling provided by Interlock Masters level counselors or	TAU (face-to-face) Interlock counselors as well	n=44 Web-based participants completed satisfaction survey,<br>n= 26 completed pre or post GAF[v] ratings	GAF increased from pre to post intervention significantly in both conditions. Effect size not specified<br><br>No group by time interaction

	Face-to-face<br>n=101<br>Age:<br>mean=44;<br>24% male			social workers			
S13. Schaub et al (2015), Germany [30]	N=308 adults with problematic cannabis use<br><br>Self help + chat: n=114<br>Age: mean 28.4 (SD 9.6); 69.3% male<br><br>Self help – chat:<br>n=101<br>Age: mean 30.2 (SD 9.2); 76.2% male<br><br>WL: n=93<br>Age: mean 29.8 (SD 10.0);<br>75.3% male	RCT	Age 18 years or older<br>Cannabis use at least weekly in the last 30 days<br><br>No history of serious psychiatric disorder<br>Not pregnant<br>Not currently undertaking other treatment for cannabis use disorder	6 weeks of self-help covering 8 modules based on principles of motivational interviewing and self-control practices with or without chat with trained counselor	WL	N=436 recruited<br><br>Self help + chat: n=87 (76%, 87/114) did not complete chat component of intervention:<br>No response: n=49;<br>Other reason: n=3;<br>No reason indicated by therapist: n=35<br><br>Follow-up: n=76 (66.6%, 76/114) no response<br><br>Self help – chat:<br>Follow-up: n=61 (60%) no response<br><br>WL:<br>Follow-up: n=55 (59%) no response	Compared with cannabis use from Swiss treatment monitoring statistics (act-info) in 2013, participants reported similar gender distribution though they tended to be older and reported higher cannabis use rates for the previous 7 days<br><br>Groups did not differ on demographic and drug use measures with the exception of number of years of cannabis use<br><br>At 3 month follow-up: mean cannabis use days/week

							differed between self-help − chat versus self-help + chat (beta= −0.75, SE[w]=0.32, *t*=−2.39, *P*=.02, d=0.34, 95% CI 0.07-0.61), and between self-help + chat versus WL (beta=.70, SE=0.32, *t*=2.16, *P*=.03, d=0.20, 95% CI −0.07 to 0.47), but not between self-help - chat versus WL (beta=−0.05, SE=0.33, *t*=−0.16, *P*=.87, d=−0.14, 95% CI −0.43 to 0.14)<br><br>No significant differences in the group comparisons in the mental health outcomes. Slight improvements in mental health (MHI-

							5[x]), Cannabis Use Disorder Identification Test, and severity of dependence scale in all 3 groups were reported (pre or post comparisons not reported)
S14. Wentz and Krevers (2012), Sweden [31]	N=12 adolescents and adults with ADHD[y] and/or an autism spectrum disorder<br><br>Age range: 15-26 years mean: 20.1 years	Naturalistic study	Aged 15-26 years Diagnosis of ASD[z] and/or ADHD, autistic disorder, Asperger disorder<br><br>Not currently diagnosed with substance abuse or dependence, depression or psychosis, low intelligence quotient	Maximum 14 session over 8-weeks Web-based support and coaching was offered at fixed times twice a week, 30 min to 1h per session		n=2 withdrew	Difference between baseline with 6-month follow-up showed significant increases on self-esteem (*P*=.02), sense of coherence (*P*=.04), and subjective global quality of life (*P*=.03)

[a]RTC: randomized controlled trial.

[b]TAU: treatment as usual.

[c]AUDIT: Alcohol Use Disorders Identification Test.

[d]CBT: cognitive behavioral therapy.

[e]WL: waitlist.

[f]TAO: Therapy Alcohol Online;

[g]SAO: Self-help Alcohol Online

[h]QOLS: Quality of Life Scale.

[i]STAI: State-Trait Anxiety Inventory.

[j]SDQ: The Strengths and Difficulties Questionnaire.

[k]YHC: youth health care.

[l]SD: standard deviation.

[m]ES: effect size.

[n]BDI: Beck Depression Inventory.

[o]GHQ-12: 12-item General Health Questionnaire.

[p]SIS: Session Impact Scale

[q]DSM-IV: Diagnostic and Statistical Manual of Mental Disorders, 4th edition.

[r]MDD: major depressive disorder.

[s]OR: odds ratio.

[t]CES-D: Center for Epidemiologic Studies Depression Scale

[u]HIV: human immunodeficiency virus.

[v]GAF: Global Assessment of Functioning.

[w]SE: standard error.

[x]MHI-5: 5-item Mental Health Inventory.

[y]ADHD: Attention-deficit/hyperactivity disorder.

[z]ASD: Autism spectrum disorder.

References

18. Blankers M, Koeter MW, Schippers GM. Internet therapy versus internet self-help versus no treatment for problematic alcohol use: a randomized controlled trial. J Consult Clin Psychol 2011 Jun; 79(3):330-341

19. Cohen GE, Kerr BA. Computer-Mediated Counseling. Computers in Human Services 1999; 15(4):13-26

20. Crutzen R, Bosma H, Havas J, Feron F. What can we learn from a failed trial: insight into non-participation in a chat-based intervention trial for adolescents with psychosocial problems. BMC Res Notes 2014 Nov 20; 7:824

21. Dowling M, Rickwood D. A naturalistic study of the effects of synchronous online chat counselling on young people's psychological distress, life satisfaction and hope. Counsell Psychother Res J 2015; 15(4):274-283

22. Fukkink RG, Hermanns JM. Children's experiences with chat support and telephone support. J Child Psychol Psychiatry 2009 Jun; 50(6):759-66

23. Fukkink R, Hermanns J. Counseling children at a helpline: chatting or calling?. J Community Psychol 2009; 37(8):939-948

24. Kessler D, Lewis G, Kaur S, Wiles N, King M, Weich S, Sharp DJ, Araya R, Hollinghurst S, Peters TJ. Therapist-delivered internet psychotherapy for depression in primary care: a randomised controlled trial. Lancet 2009 Aug 22; 374(9690):628-634

25. King R, Bambling M, Reid W, Thomas I. Telephone and online counselling for young people: a naturalistic comparison of session outcome, session impact and therapeutic alliance. Counsell Psychother Res J 2006 Sept; 6(3):175-181

26. Kordy H, Wolf M, Aulich K, Bürgy M, Hegerl U, Hüsing J, Puschner B, Rummel-Kluge C, Vedder H, Backenstrass M. Internet-delivered disease management for recurrent depression: a multicenter randomized controlled trial. Psychother Psychosom 2016; 85(2):91-8

27. Kramer J, Conijn B, Oijevaar P, Riper H. Effectiveness of a web-based solution-focused brief chat treatment for depressed adolescents and young adults: randomized controlled trial. J Med Internet Res 2014 May 29; 16(5):e141

28. Lelutiu-Weinberger C, Pachankis JE, Gamarel KE, Surace A, Golub SA, Parsons JT. Feasibility, acceptability, and preliminary efficacy of a live-chat social media intervention to reduce HIV risk among young men who have sex with men. AIDS Behav 2015 Jul; 19(7):1214-27

29. Murphy L, Parnass P, Mitchell DL, Hallett RH, Cayley P, Seagram S. Client satisfaction and outcome comparisons of online and face-to-face counselling methods. Br J Soc Work 2009 Apr 15; 39(4):627-640

30. Schaub MP, Wenger A, Berg O, Beck T, Stark L, Buehler E, Haug S. A web-based self-help intervention with and without chat counseling to reduce cannabis use in problematic cannabis users: three-arm randomized controlled trial. J Med Internet Res 2015 Oct 13; 17(10):e232

31. Wentz E, Nydén A, Krevers B. Development of an internet-based support and coaching model for adolescents and young adults with ADHD and autism spectrum disorders: a pilot study. Eur Child Adolesc Psychiatry 2012 Nov; 21(11):611-22
